# S07-2: Principles for quality design of green spaces to ensure equal hepa opportunities for all – experience from Slovenia

**DOI:** 10.1093/eurpub/ckae114.228

**Published:** 2024-09-26

**Authors:** Ina Šuklje Erjavec, Jana Kozamernik, Vita Žlender

**Affiliations:** Urban Planning Institute of the Republic of Slovenia, Ljubljana, Slovenia; Urban Planning Institute of the Republic of Slovenia, Ljubljana, Slovenia; Urban Planning Institute of the Republic of Slovenia, Ljubljana, Slovenia

## Abstract

**Purpose:**

Numerous studies have shown that green spaces are an important determinant of HEPA, in terms of promoting daily PA as well as of the greater health benefits of outdoor PA in contact with nature. However, people do not have same opportunities for PA nor the same access to green spaces. There are wide disparities between countries, cities, neighbourhoods, urban and rural environments and between different population groups. Vulnerable groups are particularly disadvantaged in this respect. In addition, not all green spaces are suitable or attractive for PA but must meet several conditions related to the type of PA and the needs and abilities of the users. For that comprehensive, inclusive, and long-term spatial planning is needed.

**Project description:**

In line with WHO recommendations and guidelines, the Ministry of Health of the Republic of Slovenia has taken a step towards cross-sectoral integration of HEPA and green space planning and in 2017 started co-funding a three-year programme entitled Expert basis for designing green spaces to promote physical activity – Going Out for Health. Currently, the Urban Planning Institute of the Republic of Slovenia, is implementing the third program: Green Spaces for Active Lifestyles, Healthy Cities, and Municipalities (https://venzazdravje.uirs.si/en-us/) , focusing mainly on practical support to local communities in the form of consultancy workshops to assess and improve the condition of their green spaces for HEPA and their accessibility for people of all ages with the special focus on vulnerable groups.

**Conclusions:**

To improve the cross-sectoral understanding of the importance of and approach to ensuring appropriate, inclusive spatial conditions for HEPA in the context of green spaces, we will present some of the results of all three programmes relating to aspects of the quality of planning and design of green spaces in relation to equitable accessibility and usability for all. We will present in more detail our eight HEPA Green Space Design Quality Principles and the methodology of the Municipal Consultations on the topic, with a particular focus on the inclusion of people with disabilities.

**Funding:**

Ministry of Health of the Republic of Slovenia, Slovenian Research and Innovation Agency, grant. no. V5-2232.

